# An Uncommon Side Effect of Rivaroxaban: A Drug-Induced Liver Injury

**DOI:** 10.7759/cureus.45949

**Published:** 2023-09-25

**Authors:** Bhesh R Karki, Ranjit B Jasaraj, Suman K Jha, Nodari Maisuradze, Qi Yu

**Affiliations:** 1 Internal Medicine, State University of New York (SUNY) Downstate Health Sciences University, New York City, USA; 2 Internal Medicine, Mount Sinai Hospital Medical Center of Chicago, Chicago, USA; 3 Family Medicine, University of North Dakota, Fargo, USA; 4 Gastroenterology and Hepatology, State University of New York (SUNY) Downstate Health Sciences University, New York City, USA

**Keywords:** deranged liver function tests, elevated transaminases, direct oral anticoagulant, hepatotoxicity, drug induced liver injury, rivaroxaban

## Abstract

Rivaroxaban is rarely associated with drug-induced liver injury (DILI). A 57-year-old male was sent to the emergency room from an endocrine clinic for a presyncope evaluation. His exam was non-focal, and his laboratory work was remarkable for the hepatocellular pattern of liver injury. Upon detailed assessment, he was found to have DILI due to rivaroxaban. The liver function tests improved after its discontinuation. This case emphasizes the need for early recognition and timely intervention to prevent further hepatotoxicity from the culprit drug.

## Introduction

Rivaroxaban is a commonly used direct oral anticoagulant (DOAC) and works by inhibiting factor Xa. Its major uses include prophylaxis and treatment of venous thromboembolism, stroke prevention in atrial fibrillation, and an add-on drug for the secondary prevention of acute coronary syndrome and peripheral arterial disease [1]. Direct oral anticoagulants like rivaroxaban do not require monitoring of anticoagulation effects, unlike warfarin. Bleeding (major or minor) is the most common adverse reaction associated with rivaroxaban [2]. Although hepatotoxicity is a known side effect of rivaroxaban, it is rare considering its widespread use. We present a case of liver injury due to rivaroxaban.

## Case presentation

A 57-year-old African American male with a history of Graves’ disease, heart failure with reduced ejection fraction (HFrEF), and recent hepatitis B reactivation was sent to the emergency room (ER) from the endocrine clinic for presyncope. He had visited the endocrine clinic for regular follow-up after being discharged from the hospital about a week prior. In the clinic, he reported feeling weak, tired, and dizzy. His vital signs were significant, with a blood pressure of 78/54 mmHg. He was then sent to the ER for further evaluation.

In the ER, his repeat blood pressure was 76/57 mmHg. His mucus membranes were moist, and he did not have pallor, icterus, or lymphadenopathy. The remainder of his physical examination was unremarkable. His pertinent laboratory results included thyroid stimulating hormone (TSH): < 0.01 µIU/mL, free thyroxine (fT4): 2.48 ng/dL, alanine aminotransferase (ALT): 991 U/L, aspartate aminotransferase (AST): 623 U/L, alkaline phosphatase (ALP): 158 U/L, total bilirubin (T bili): 2.1 mg/dL, and prothrombin time/international normalized ratio (PT/INR): 13.9s/1.2. The right upper quadrant sonogram showed a benign hepatic cyst along with normal liver parenchyma without any intrahepatic or extrahepatic bile duct dilatation.

Thirteen days before this presentation, he was admitted to the hospital for atrial fibrillation with rapid ventricular response secondary to thyrotoxicosis due to non-compliance with methimazole. Pertinent laboratory results during that admission included TSH: <0.01 µIU/ml, fT4: 4.32 ng/dL, T bili: 1.6 mg/dL, ALP: 150 U/L, ALT: 427 U/L, and AST: 163 U/L. Laboratory workup for autoimmune causes of liver injury, including antinuclear antibody, anti-smooth muscle antibody, antimitochondrial antibody, liver-kidney microsomal antibody, immunoglobulin panel, cytomegalovirus, herpes simplex virus, and Epstein-Barr virus immunoglobulin M (IgM), was negative. However, his hepatitis panel revealed a positive hepatitis B surface antigen, positive hepatitis B core IgM and total antibody, positive hepatitis B surface antibody, negative hepatitis B e antigen, a viral load of 1.5 million IU/mL, negative hepatitis C antibody, negative hepatitis A IgM, positive hepatitis A total antibody, and negative hepatitis D antibody. He was started on methimazole, propranolol, sacubitril/valsartan, empagliflozin, rivaroxaban, and tenofovir.

**Figure 1:**
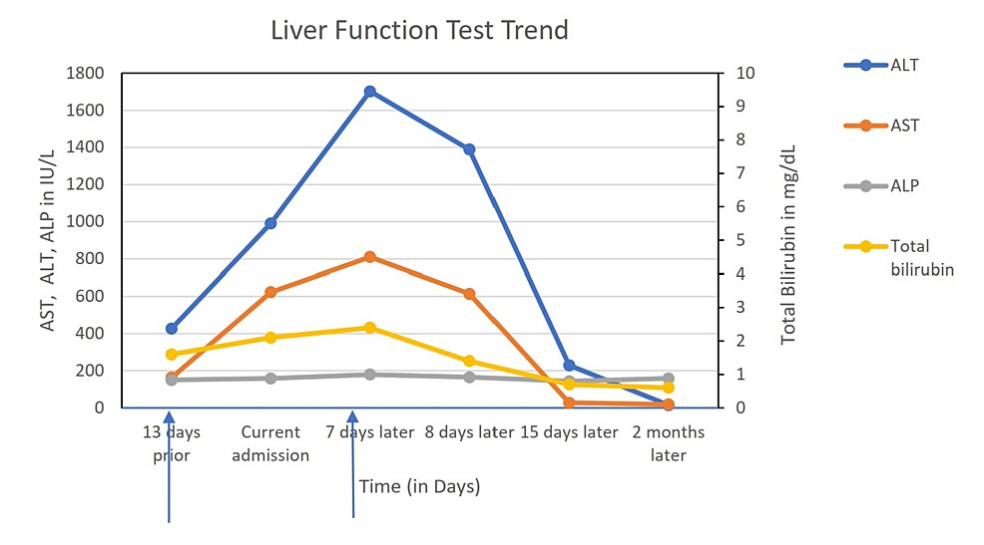
A line diagram depicting the values of LFTs on different occasions (the first vertical arrow denotes rivaroxaban initiation; the second vertical arrow denotes rivaroxaban discontinuation) LFT: liver function test; AST: aspartate aminotransferase; ALT: alanine aminotransferase; ALP: alkaline phosphatase

While in the emergency department, he received a liter bolus of intravenous crystalloid, resulting in an improvement of his blood pressure to 104/68 mmHg. He was then admitted for further care. The sacubitril/valsartan was discontinued on admission, and the rest of his home medications were continued. While in the hospital, his liver function tests (LFTs) continued to uptrend for the next seven days (Figure 1).

Rivaroxaban was discontinued on the sixth day, and apixaban was started. His LFTs started to downtrend on the eighth day of admission (Figure 1). The hospital stay was otherwise uneventful. He was then discharged on the tenth day of admission. Follow-up at two weeks showed significant improvement in liver enzymes, with normal enzymes at two months (Table 1).

**Table 1:** Laboratory values of LFTs on different days LFT: liver function test; AST: aspartate aminotransferase; ALT: alanine aminotransferase; ALP: alkaline phosphatase; T bili: total bilirubin

Lab parameters	13 days prior	Current admission	7 days later	8 days later	15 days later	2 months later
ALT	427	991	1702	1389	229	15
AST	163	623	810	614	27	18
ALP	150	158	179	165	143	158
T bili	1.6	2.1	2.4	1.4	0.7	0.6

## Discussion

Drug-induced liver injury is common, with an incidence of about 2.7 per 100,000 exposed cases, and occurs with nearly all classes of medications [3]. Drug-induced liver injury is the most common cause of acute liver failure in Western countries [4]. The diagnosis of DILI is challenging due to the lack of specific biomarkers for drug-induced hepatotoxicity. So, the assessment for DILI includes ruling out all other probable causes of the liver injury [5]. In our case, we ruled out multiple probable explanations for elevated transaminases including thyrotoxicosis, hepatitis B, and ischemia. Although he had hyperthyroidism, he was already taking antithyroid drugs, and his repeat thyroid function test was improving. Similarly, the patient was already on tenofovir prior to admission, making hepatitis B reactivation the unlikely culprit in his progressive derangement of LFTs on this admission. His blood pressure was low at the presentation, but it was transient and improved with a fluid bolus. Given only the transient drop in blood pressure and the degree of elevation in the liver enzymes, ischemia was less likely to be the etiology as well. The calculated Roussel-Uclaf causality assessment method (RUCAM) score was eight in our case, indicating rivaroxaban as the “probable” cause of DILI [6]. Taking the whole picture into consideration, a preliminary diagnosis of DILI due to rivaroxaban was made, and rivaroxaban was switched to apixaban on the sixth day. The liver enzymes started to trend down on the eighth day, as shown in the graph, further supporting our diagnosis of DILI due to rivaroxaban.

Drug-induced liver injury due to DOACs is rare, with a frequency of 0.1%-1% [7]. Although rivaroxaban has been associated with a higher incidence of DILI among DOACs [8], it is still an unusual manifestation considering its most widespread use. In a recent systematic review analyzing the hepatotoxic effects of rivaroxaban, jaundice was the most commonly reported symptom, followed by malaise and vomiting [9]. Our patient had malaise but no jaundice or vomiting. The most common pattern of liver injury from rivaroxaban is hepatocellular, followed by cholestatic and mixed patterns, respectively [10]. Our patient had a hepatocellular pattern of liver injury with an R-factor of 18.8. Rivaroxaban has a dual mode of elimination: one-third is excreted via the kidneys, and two-thirds is metabolized by the liver. It is metabolized by the cytochrome CYP450 (CYP) 3A4 isoform, and hence, its efficacy is affected by CYP3A4 inducer and inhibitor drugs [11]. This patient was not taking any of the inhibitor drugs that could potentiate the toxicity of rivaroxaban.

The median time between rivaroxaban initiation and liver injury is 15 days [10]. This patient was taking rivaroxaban for 13 days at his presentation. Although not clearly established, the mechanism of liver injury from rivaroxaban is likely idiosyncratic and possibly immunologic [12]. Human leukocyte antigen (HLA) genotypes are also being increasingly linked to elevated DILI risk [12]. Most cases of DILI are benign and improve after the removal of the offending agent. So is the hepatotoxicity of rivaroxaban. Rarely, it has been described to cause acute liver failure as well [13], along with death, as a very unusual occurrence [9]. Given the wide use of rivaroxaban, this case aims to serve as a reminder to consider rivaroxaban as a cause of DILI and switch to an alternative agent in a timely manner.

## Conclusions

Rivaroxaban is a widely prescribed DOAC as a prophylaxis and treatment for several conditions. Although DILI due to DOACs is rare, rivaroxaban has been associated with a higher incidence of liver injury compared to other DOACs. Diagnosing DILI can be challenging, but it is important to consider this in patients with abnormal liver function tests. The temporal association of rivaroxaban initiation and deranged LFTs, along with ruling out other etiologies and the RUCAM score, help in making a diagnosis of DILI from rivaroxaban. This case highlights rivaroxaban as a potential cause of DILI. Prompt intervention can help prevent potential liver failure and the need for more aggressive treatment like liver transplantation.
